# Eating Disorders and Intimate Partner Violence: The Influence of Fear of Loneliness and Social Withdrawal

**DOI:** 10.3390/nu14132611

**Published:** 2022-06-24

**Authors:** Janire Momeñe, Ana Estévez, Mark D. Griffiths, Patricia Macía, Marta Herrero, Leticia Olave, Itziar Iruarrizaga

**Affiliations:** 1 Psychology Department, School of Health Sciences, University of Deusto, 48080 Bilbao, Spain; janiremomene@deusto.es (J.M.); aestevez@deusto.es (A.E.); m.herrero@deusto.es (M.H.); 2 International Gaming Research Unit, Psychology Department, Nottingham Trent University, Nottingham NG1 4FQ, UK; mark.griffiths@ntu.ac.uk; 3 Department of Basic Psychological Processes and Their Development, University of the Basque Country/Euskal Herriko Unibertsitatea (UPV/EHU), 20018 Donostia-San Sebastián, Spain; patricia.macia@ehu.eus; 4 Department of Experimental Psychology, Cognitive Processes and Speech Therapy, Faculty of Social Work, Complutense University of Madrid, Pozuelo de Alarcón, 28223 Madrid, Spain; leticiaolave@ucm.es

**Keywords:** eating disorders, intimate partner violence, violence received, social withdrawal, fear of loneliness, vulnerability factors, path analysis

## Abstract

Eating disorders are vulnerability factors that increase the likelihood of intimate partner violence. However, the mechanisms underlying this relationship are unclear. Although eating disorders have been associated with increased perception and fear of loneliness, they have also been associated with increased social withdrawal resulting from decreased enjoyment of social situations and poorer social functioning. The purpose of the present study was to examine the mediating role of fear of loneliness in the relationship between the behavioural characteristics of eating disorders and intimate partner violence, as well as to explore the moderating role of social withdrawal in the relationship between fear of loneliness and intimate partner violence. The sample comprised 683 participants (78% female and 22% male) with a mean age of 21.14 years (SD = 2.72). The psychometric scales used were Eating Disorders Inventory (EDI 2), Emotional Dependency Questionnaire (EDQ), Coping Strategies Inventory (CSI) and the Violence Received, Exercised and Perceived in Youth and Adolescent Dating Relationships Scale (VREPS). The hypothesised model was tested by path analysis using maximum likelihood. The path analysis of the hypothesised model showed that inefficacy, fear of maturity, and impulsivity were the behavioural characteristics of eating disorders predominantly related to fear of loneliness. Fear of loneliness had no direct significant effect on any of the received violence variables. However, interaction effects indicated that there was a moderately significant effect of fear of loneliness on physical, psychological, and social violence received as a function of levels of social withdrawal. These findings show the need to take into account and work on fear of loneliness and social withdrawal among individuals with an eating disorder to decrease the likelihood of establishing violent intimate partner relationships. Improving interpersonal functioning and social support is key to recovery from eating disorders.

## 1. Introduction

Eating Disorders (EDs) are serious psychiatric disorders [1] that significantly impair the physical and psychological health of sufferers. Moreover, the mortality rate is one of the highest compared to other psychiatric conditions (5–10%) [2,3]. Currently, the main types of EDs that individuals suffer from worldwide are anorexia nervosa, bulimia nervosa, and binge eating disorder [4], characterised by persistent disturbance of eating behaviour [5]. It has been estimated that EDs affect 15% of the world population and their incidence continues to increase. Moreover, they begin to manifest themselves between early and late adolescence. It is at this stage of the life cycle that important physical, psychological and neuronal changes occur [6]. Its aetiology is multifactorial, and psychological, developmental, biological and/or sociocultural factors may influence it. However, the aetiology is not yet fully elucidated. In recent years, the need for further research has been noted [2,7]. 

In addition to the deterioration produced in physical and psychological health, EDs can also negatively impacts social functioning [8,9,10,11]. The perception of loneliness has been found to be present in individuals with this problem, and it is considered a negative emotion that contributes to and increases their symptomatology. Moreover, EDs also exacerbate feelings of loneliness [12]. Perceived loneliness is defined as emotional distress stemming from a feeling of rejection or isolation by others or the lack of a social partner to lean on and engage in activities with. Moreover, it has been shown to severely influence individuals’ quality of life [13,14]. 

Recent research has highlighted the feeling of loneliness as one of the most commonly present issues among young adults [15]. However, very few studies have examined the feeling of loneliness in emerging adulthood [16] and even fewer in Spain (where the present study was carried out) in relation to EDs. Understanding the relationship between EDs and fear of loneliness is vital to address these intense emotions within prevention and treatment programs. Therefore, it is important to analyse the impact exerted by the fear of loneliness among individuals with this problem [12,17]. 

In addition, previous studies have noted the use of dysfunctional coping strategies by individuals with an ED [18,19] that contribute to the aetiology and maintenance of this problem [6,20]. Therefore, the behaviours characteristic of EDs can be employed as dysfunctional coping mechanisms to regain control over stressful circumstances [21]. More specifically, findings suggest that individuals with an ED predominantly employ coping strategies based on self-criticism and social isolation. Furthermore, the importance of further research has been pointed out because coping strategies play an important role in the prognosis and treatment of EDs [22], especially social isolation. Empirical studies suggest that social isolation and low sense of social support increase ED symptomatology and have a detrimental impact on recovery [23,24,25,26]. This may be because social isolation promotes increased maladaptive eating habits and body dissatisfaction [27]. Therefore, it has been noted that social support and adaptive social functioning are key to a more effective and complete recovery [28,29].

Likewise, the empirical literature has noted that EDs increase the probability of suffering intimate partner violence (IPV) throughout life [30]. Therefore, the prevalence of IPV among individuals with EDs is high [31]. It should be noted that previous literature has also posited a bidirectional relationship between IPV and EDs because the direction of causality can be in both directions [32]. However, the mechanisms underlying this relationship are unclear. Previous studies have found that social isolation and fear of loneliness are vulnerability factors for staying in violent relationships [33]. Despite this, the role they play in the relationship between EDs and IPV has not been established. Consequently, their study is of utmost importance in designing early and effective prevention and intervention programs [34]. 

In recent years, this line of research examining the relationship between EDs and IPV has gained relevance due to its clinical and prognostic implications. Therefore, the present study’s main objectives were to: (i) analyse the relationships between core symptoms traversing Eds; (ii) explore the mediating role of fear of loneliness in the relationship between the behavioural characteristics of EDs and IPV; and (iii) explore the moderating role of social isolation in the relationship between fear of loneliness and IPV. Based on the aforementioned literature, the hypotheses of the present study were that: (i) the core symptoms traversing EDs will have a significant direct effect on received partner violence; and (ii) the core symptoms traversing EDs will have a significant indirect effect on received partner violence through the mediating role of fear of loneliness and the moderating role of social isolation.

## 2. Method

### 2.1. Participants

The sample comprised 683 emerging Spanish adults who participated in a cross-sectional survey study. The average age of the participants was 21.14 years old (SD = 2.72; 78% female and 22% male). The participants were mostly students (80.1%) and workers (19.3%). The remaining participants were unemployed (0.6%). 

### 2.2. Procedure

Participants were recruited through two channels: online and face-to-face. For the online recruitment, surveys were made available through an online platform (*surveymonkey.com* accessed on 1 January 2020). Participation was promoted through different social networks and advertisements on research websites. For the face-to-face recruitment, participants were recruited at the Complutense University of Madrid and at gyms in the Madrid community. The only exclusion criterion was being under 18 years of age. All participants gave their informed consent by confirming or clicking on a button indicating that they had read the study information and agreed to participate voluntarily. The study followed the ethical principles of the 2013 Helsinki Declaration and was approved by the research team’s university ethics committee.

### 2.3. Instruments

*Eating disorder characteristics.* The Eating Disorders Inventory-2 (EDI-2) [35] was used to assess clinically relevant behaviours and psychological traits that accompany EDs. The EDI-2 consists of 91 items divided into 11 scales (obsession with thinness, bulimia, body dissatisfaction, inefficacy, perfectionism, interpersonal distrust, interoceptive awareness, fear of maturity, asceticism, impulsivity and social insecurity). All items (e.g., *“I tend to eat when I am upset”*; *“I find it difficult to express my emotions to others”*; *“I think my stomach is too big”*) are rated on a six-point scale from 0 (*“Never”*) to 5 (*“Always”*). The higher the scores obtained on each scale, the greater the manifestations of the trait evaluated. The internal consistency (Cronbach’s α) of the subscales in the present study ranged from 0.73 to 0.90.

*Fear of loneliness.* The fear of loneliness subscale from the Emotional Dependency Questionnaire (EDQ) [36] was used to assess fear of loneliness. All items (e.g., *“I feel helpless when I am alone”*; *“I feel a strong sense of emptiness when I am alone”*; *“I cannot tolerate loneliness”*) are rated on a six-point scale from 1 (*“Completely untrue of me”*) to 6 (*“Describes me perfectly”*). The higher the score obtained, the greater the fear of loneliness. The internal consistency in the present study was α = 0.82.

*Social avoidance.* The Coping Strategies Inventory (CSI) [37] was used to assess social avoidance. The scale assesses eight styles of coping with stressful situations by means of 41 items (problem solving, cognitive restructuring, social support, emotional expression, problem avoidance, desiderative thinking, social withdrawal, self-criticism). All items (e.g., *“I avoided being with people”*; *“I didn’t let anyone know how I felt”*) are rated on a five-point scale from 0 (*“Not at all”*) to 4 (*“Completely”*). The higher the score obtained, the greater the social avoidance. The internal consistency in the present study was α = 0.74.

*Received violence.* The Violence Received, Exercised and Perceived in Youth and Adolescent Dating Relationships Scale (VREPS) [38] was used to assess received violence. The scale comprises 28 items including five violence subscales (physical violence, sexual violence, social psychological violence, psychological violence humiliation–coercion, and psychological violence control-jealousy) and encompassing three aspects of violence: received, exerted, and perceived. For violence received and exercised, items (e.g., *“My boyfriend/girlfriend tells me to change the way I dress, do my hair… and criticizes it”*; *“My boyfriend/girlfriend wants to know where I am at all times and who I am with”*; *“My boyfriend/girlfriend has run out of friends because I didn’t like them and told him/her not to be with them”*) are rated on a six-point scale (0 *“Never”,* 1 *“Once”,* 2 *“From 2 to 5 times”,* 3 *“From 6 to 10 times”,* 4 *“From 11 to 15 times”* and 5 *“More than 15 times”*) and for perceived violence items (e.g., *“My boyfriend/girlfriend has forced me to have sex (any kind of oral or penetration) when I did not want to. Is this violence?”*) are rated on a five-point scale (1 *“No violence”,* 2 *“Little violence”,* 3 *“Somewhat violent”,* 4 *“Quite violent”* and 5 *“Very violent”*). In addition, participants indicate whether they consider the situations mentioned to be violence. The higher the score obtained, the greater the received violence. In the present study, the violence received was of particular interest in the analysis. The internal consistency of the five subscales of received violence in the present study ranged from α = 0.82 to 0.89.

### 2.4. Statistical Analysis

**Figure 1:**
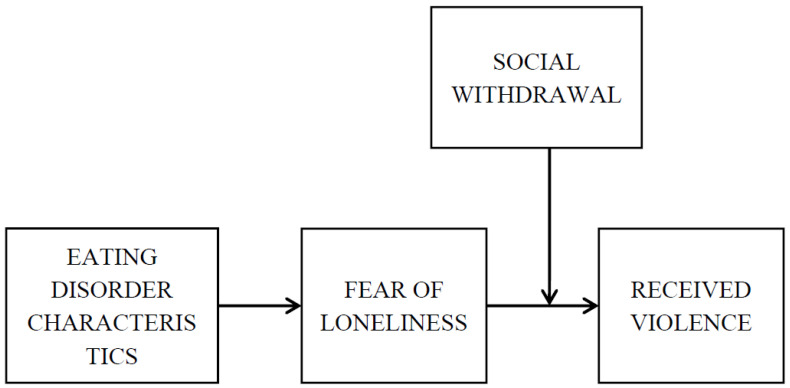
Hypothesised model.

Data analyses were carried out using Mplus 7.0 [39]. The hypothesised model was tested by path analysis using maximum likelihood. Following the model described in Figure 1, the model included the eating disorder characteristics (i.e., obsession for thinness, bulimia, body dissatisfaction, ineffectiveness, perfectionism, interpersonal distrust, interoceptive awareness, fear of maturity, asceticism, impulsiveness and social insecurity) as independent variables, received violence as the dependent variable (i.e., physical, sexual, psychological humiliation–coercion, psychological control-jealousy and social), the fear of loneliness as the mediator, and social withdrawal as the moderator in the relationship between the mediator and the dependent variables. Gender and age were included as controls in the model.

The adequacy of the proposed model was analysed according to the following model fit indicators: ratio of chi-square (χ^2^) and the degrees of freedom, the comparative fit index (CFI), the Tucker–Lewis index (TLI), the root mean squared error of approximation (RMSEA), and the standardised root mean square residual (SRMR). Values of χ^2^/df of <3.0, CFI and TLI ≥ 0.90, and RMSEA and SRMR < 0.08 were considered indicators of good fit [40].

In order to test the moderated mediation, the direct effect of EDCs (eating disorder characteristics) on the dependent variables was included, and the variables of the products were standardised. Additionally, the analysis adapted the code provided by Stride et al. (2015) [41] in Model 1 and Model 14 to test the simple slopes of the direct and indirect effects. For the computation of the indirect effects, bootstrap was applied with 5000 samples. All significant moderations and moderated-mediations were tested at low (−1.5 SD), average (at the mean) and high levels (+1.5 SD) of the moderator to examine simple slopes. 

## 3. Results

First, the descriptive statistics of the sample and the correlations between the study variables were calculated (see Table 1 and Table 2). Some of the EDCs were not significantly correlated with any of the received violence indicators (i.e., bulimia, body dissatisfaction and fear of maturity). Fear of loneliness and social withdrawal were significantly correlated with all variables.

Second, the path analysis of the hypothesised model was performed. All model fit indicators showed a good fit of the model, χ^2^/df = 2.26, CFI = 0.99, TLI = 0.95, RMSEA = 0.04, SRMR < 0.01. Therefore, the model adequately explained the study data. As displayed in Table 3, the direct effects showed that ineffectiveness, fear of maturity, and impulsiveness were the EDCs related to fear of loneliness. Fear of loneliness had no direct significant effect on any of the variables of received violence. However, the interaction effects indicated that there was a significant moderated effect of fear of loneliness on physical, psychological humiliation–coercion, and social received violence depending on the levels of social withdrawal.

Based on the exposed variables, the simple slopes were examined to understand the moderation effects. Simple slopes showed that higher fear of loneliness was significantly related to more physical violence (*β* = 0.16, SE = 0.03, *p* < 0.001) and psychological humiliation–coercion received violence (*β* = 0.11, SE = 0.05, *p* = 0.028) when the social withdrawal was high, but there was no significant relationship when the social withdrawal was low (physical: *β* = −0.07, SE = 0.03, *p* = 0.063; psychological humiliation––coercion: *β* = −0.06, SE = 0.05, *p* = 0.281) or medium (physical: *β* = 0.16, SE = 0.03, *p* = 0.053; psychological humiliation––coercion: *β* = 0.02, SE = 0.03, *p* = 0.444).

Regarding social received violence, the simple slopes indicated that greater fear of loneliness was significantly related to lower social received violence when the social withdrawal was low (*β* = −0.19, SE = 0.04, *p* = 0.043), but was related to greater social violence when the social withdrawal was high (*β* = 0.13, SE = 0.04, *p* = 0.002). The relationship at medium levels of social withdrawal was not significant (*β* = 0.01, SE = 0.02, *p* = 0.499).

As a final step, the moderated mediation of the EDCs was tested on the variables of received violence (see Table 4). The indirect effects of ineffectiveness, fear of maturity and impulsiveness on physical violence, and social received violence, through fear of loneliness, were significant only at high social withdrawal levels. However, none of the indirect effects on psychological humiliation–coercion received violence was significant, although a tendency was observed towards high social withdrawal levels.

## 4. Discussion

The main objective of the present study was to analyse the association between core symptoms traversing eating disorders (EDs) and to explore the role of fear of loneliness and social isolation in relation to behavioural eating disorder characteristics (EDCs) and intimate partner violence (IPV) received. First, it was hypothesised that core symptoms traversing EDs would have a significant direct effect on received partner violence. Results showed that some of the EDCs were not significantly associated with any of the received violence indicators (e.g., bulimia, body dissatisfaction, and fear of maturity). However, other indicators such as obsession for thinness, ineffectiveness, perfectionism, interoceptive awareness, asceticism, impulsiveness, and social insecurity were all significantly and positively related to received violence. 

These results are in accordance with previous scientific literature that EDs increase the likelihood of IPV among both females and males [30,31]. Although EDs have traditionally been considered female disorders, recent evidence suggests that it is not uncommon among males, and that males can present similar severe ED symptoms. In fact, there are specific risk factors for developing EDs among young and adolescent males, such as body image concerns related to muscularity and sexual orientation [1]. As mentioned, EDs can emerge as maladaptive coping mechanisms that enable individuals to regain control over adverse situations, as can be receiving violence [21,42].

Another factor associated with EDs and violence exposure among both sexes is social isolation, which has been associated with adoption of unhealthy weight control practices [26]. With the aim of exploring more deeply the role of social aspects, the second hypothesis was that core symptoms traversing EDs would have a significant indirect effect on received partner violence through the mediating role of fear of loneliness and the moderating role of social isolation. 

On the one hand, results showed that ineffectiveness, fear of maturity, and impulsivity were the behavioural EDCs predominantly related to fear of loneliness. Fear of loneliness had no direct significant effect on any of the received violence variables. Nevertheless, interaction effects indicated a moderately significant influence of fear of loneliness on physical violence, psychological humiliation—coercion, and social received violence as a function of levels of social withdrawal. 

It was also found that the indirect effects of ineffectiveness, fear of maturity, and impulsiveness on physical and social received violence, through fear of loneliness, were significant only at high social withdrawal levels. Results refine the understanding of the relationship between social withdrawal and the development of EDs in individuals exposed to partner violence. Individuals suffering loneliness appear to be more susceptible to developing disordered eating patterns [43]. 

Participants in the present study are characterised as being young. Moreover, it should be noted that emerging adulthood can be a critical period for developing mental health problems [15,16]. In particular, EDs are frequently initiated in this period, especially, considering the great relevance that acquire social interactions at this developmental stage [18]. Fear of loneliness and social isolation are among the most common concerns for young people, and results have evidenced their impact on the relation between EDs and IPV [15].

For instance, the pandemic and subsequent social restrictions have limited and deprived individuals of social interaction resulting in decreased social support and similar coping strategies in facing this unprecedented situation [44]. Therefore, eliminating social protection factors when coping with adverse events could increase risk and symptoms of ED [45]. In this sense, loneliness has been conceived as a mediator between emotional dysregulation and eating disorders-related psychopathology [46]. 

This lack of perceived social support associated with the exposure to partner violence could culminate in many psychological health consequences, such as depression, post-traumatic stress, anxiety, and EDs, among other mental health illnesses [47]. Low levels of social support have been related to increased risk of ED among women exposed to IPV. Social support has shown protective effects against ED by decreasing levels of anxiety and promoting mechanisms related to functional coping strategies [48]. However, IPV-exposure and trauma history can precede the development of ED symptoms. The extant literature highlights the presence of childhood abuse among individuals suffering IPV and EDs. Children who have experienced exposure to violent situations appear to be more susceptible to developing EDs [30,49]. Other studies have identified specific aspects related to altered-eating behaviours and IPV exposure including somatization, avoiding abuse, coping, self-harm, and challenging abusive partners [42].

Overall, results in the present study confirm the bidirectional relationship between ED and IPV, influenced by aspects such as fear of loneliness and social withdrawal. On the one hand, it was observed that childhood abuse is highly related to both EDs and IPV, so it could be considered a possible explanatory factor [32,49,50,51]. On the other hand, it should be noted that the association between EDs and IPV also depends on the type of ED, due to the fact that different EDs have diverse aetiology [52]. Nevertheless, the present study highlights social-related aspects in explaining some of the mechanisms underlying this bidirectional relationship between EDs and IPV. Individuals who suffer from EDs usually show fear of loneliness and social isolation patterns, which are also consequences of IPV, and could likewise derive in developing ED-related symptoms. 

All of these aspects support the notion that ED-related behaviours are used as ways to cope with adverse and stressful situations such as received violence. This could be important information for therapists who work with those experiencing IPV and who develop interventions for patients with clinical symptoms of an ED. Results emphasise the importance of understanding the vulnerability and absence of coping resources among individuals who suffer IPV and develop EDs, with the aim of designing interventions focused on the promotion of coping through seeking social support and avoiding isolation. 

## 5. Limitations

The present study has some limitations that should be noted. First, the cross-sectional design employed in the present study does not allow determining conclusions in terms of causality. Therefore, longitudinal studies are needed to determine any casual inferences among different variables examined in the present study. Secondly, the sample in the present study was limited to emerging adults, with an average age of 21 years old, therefore results cannot necessarily be generalised to other age groups. 

In future research, it would be interesting to extend the study to other age populations, with the aim of exploring differences in ED-behaviour patterns and IPV related to social isolation aspects in other developmental phases. In addition, the present study did not explore differences by sex in the variables of interest. Efforts to increase the number of male participants would be of utility with the objective of homogenising the sample and analysing differences in ED patterns and different symptoms related to received violence in relationships. 

## 6. Conclusions

Eating disorders are vulnerability factors that increase the likelihood of intimate partner violence. Nevertheless, the mechanisms underlying this relationship are unclear. The present study has explored the influence of withdrawal as a result of decreased enjoyment of social situations and poorer social functioning. Overall, the results of the present study demonstrate the role of social-related aspects in the relationship between EDs and IPV. 

It is suggested that individuals exposed to violent situations in relationships may develop ED-related symptoms as a way of coping with adverse situations. However, this relationship is not direct, and it appears that underlying mechanisms related to fear to loneliness and social withdrawal prevent the developing of coping resources for facing received violence. These findings highlight the significance of working on fear of loneliness and social withdrawal among individuals with an ED to decrease the likelihood of establishing violent intimate partner relationships. 

Future research should focus on finding ways of empowering victims through increasing social support and promoting resilience and adaptive coping resources as ways to reduce exposure to violent situations. Improving interpersonal functioning and social support is key to recovery from eating disorders.

## Figures and Tables

**Table 1:** Descriptive statistics and reliability of the study variables (n = 683).

Variables	Means and Standard Deviations				Reliability		
	M	SD	Min	Max	α	CR	AVE
Eating disorder characteristics							
*Obsession for thinness*	13.68	9.30	0	35	0.90	0.93	0.64
*Bulimia*	9.10	6.08	0	32	0.80	0.85	0.46
*Body dissatisfaction*	20.39	7.18	2	41	0.89	0.91	0.54
*Ineffectiveness*	15.14	8.78	0	48	0.88	0.90	0.48
*Perfectionism*	11.60	5.67	0	30	0.73	0.82	0.43
*Interpersonal distrust*	11.92	5.97	0	32	0.73	0.82	0.41
*Interoceptive awareness*	17.74	8.76	0	45	0.84	0.88	0.43
*Fear of maturity*	19.18	6.43	2	40	0.73	0.81	0.35
*Asceticism*	12.31	6.20	0	40	0.73	0.81	0.36
*Impulsiveness*	14.44	8.44	0	55	0.82	0.86	0.36
*Social insecurity*	13.84	6.51	0	40	0.79	0.84	0.41
Fear of loneliness	5.53	2.99	2	18	0.82	0.90	0.74
Social withdrawal	5.80	4.11	0	20	0.74	0.83	0.50
Received violence							
*Physical*	0.21	0.55	0	5	0.82	0.89	0.61
*Sexual*	0.33	0.73	0	5	0.88	0.91	0.64
*Psychological humiliation–coercion*	0.41	0.82	0	5	0.88	0.91	0.63
*Psychological control-jealousy*	0.60	0.96	0	5	0.89	0.92	0.65
*Social*	0.28	0.67	0	4.6	0.85	0.90	0.65

Note. Min = Minimum; Max = Maximum; α = Cronbach’s alpha; CR = Composite reliability; AVE = Average variance extracted.

**Table 2:** Bivariate correlations between the study variables.

Variables	Correlations																	
	1	2	3	4	5	6	7	8	9	10	11	12	13	14	15	16	17	18
Eating disorder characteristics																		
1. Obsession for thinness	(0.90)																	
2. Bulimia	0.41 **	(0.80)																
3. Body dissatisfaction	0.58 **	0.32 **	(0.89)															
4. Ineffectiveness	0.45 **	0.39 **	0.35 **	(0.88)														
5. Perfectionism	0.27 **	0.39 **	0.15 **	0.26 **	(0.73)													
6. Interpersonal distrust	0.15 **	0.14 **	0.17 **	0.53 **	0.10 **	(0.73)												
7. Interoceptive awareness	0.49 **	0.56 **	0.38 **	0.65 **	0.39 **	0.46 **	(0.84)											
8. Fear of maturity	0.23 **	0.17 **	0.19 **	0.35 **	0.20 **	0.26 **	0.40 **	(0.73)										
9. Asceticism	0.44 **	0.57 **	0.34 **	0.49 **	0.48 **	0.17 **	0.60 **	0.25 **	(0.73)									
10. Impulsiveness	0.35 *	0.52 **	0.29 **	0.52 **	0.39 **	0.24 **	0.67 **	0.28 **	0.67 **	(0.82)								
11. Social insecurity	0.21 **	0.20 **	0.20 **	0.66 **	0.13 **	0.67 **	0.43 **	0.26 **	0.27 **	0.38 **	(0.79)							
12. Fear of loneliness	0.22 **	0.26 **	0.20 **	0.37 **	0.23 **	0.16 **	0.34 **	0.25 **	0.35 **	0.38 **	0.24 **	(0.82)						
13. Social withdrawal	0.19 **	0.23 **	0.17 **	0.39 *	0.22 **	0.42 **	0.38 **	0.21 **	0.31 **	0.30 **	0.43 **	0.19 **	(0.74)					
Received violence																		
14. Physical	0.05	0.04	−0.03	0.04	0.06	0.01	0.10 **	0.07	0.09 *	0.11 **	0.05	0.12 **	0.10 **	(0.82)				
15.Sexual	0.13 **	0.04	0.02	0.11 **	0.09 *	0.04	0.11 **	0.01	0.12 **	0.15 **	0.09 *	0.09 *	0.11 **	0.53 **	(0.88)			
16. Psychological humiliation–coercion	0.07 *	0.03	0.01	0.09 **	0.10 **	0.03	0.07 *	0.02	0.12 **	0.14 **	0.10 **	0.09 *	0.08 *	0.64 **	0.67 **	(0.88)		
17. Psychological control-jealousy	0.10 **	0.06	0.03	0.08 *	0.11 **	0.04	0.11 **	0.03	0.14 **	0.19 **	0.09 *	0.11 **	0.12 **	0.57 **	0.64 **	0.81 **	(0.89)	
18. Social	0.08 *	0.03	0.03	0.12 **	0.07	0.08 *	0.10 **	0.02	0.11 **	0.13 **	0.15 **	0.09 *	0.11 **	0.60 **	0.65 **	0.86 **	0.81 **	(0.85)

Note. * *p* < 0.05, ** *p* < 0.01.

**Table 3:** Standardised direct and interaction effects of the model based on path analysis.

	Dependent Variables											
	Received Violence											
Independent Variables	Fear of Loneliness		Physical		Sexual		Psychological Humiliation–Coercion		Psychological Control-Jealousy		Social	
	*β*	SE	*β*	SE	*β*	SE	*β*	SE	*β*	SE	*β*	SE
Eating disorder characteristics												
*Obsession for thinness*	−0.03	0.04	0.08	0.05	0.14 **	0.05	0.06	0.05	0.10 *	0.05	0.06	0.05
*Bulimia*	0.02	0.04	−0.06	0.04	−0.08	0.05	−0.08	0.05	−0.09 *	0.04	−0.08	0.05
*Body dissatisfaction*	0.05	0.04	−0.12 *	0.04	−0.09 *	0.04	−0.05	0.04	−0.04	0.04	0.04	0.04
*Ineffectiveness*	0.20 ***	0.05	−0.12	0.06	0.01	0.06	<−0.01	0.06	−0.08	0.06	<0.01	0.06
*Perfectionism*	0.05	0.04	<0.01	0.04	0.04	0.04	0.06	0.04	0.04	0.04	0.03	0.04
*Interpersonal distrust*	−0.03	0.04	−0.08	0.05	−0.03	0.05	−0.04	0.05	−0.02	0.05	−0.03	0.05
*Interoceptive awareness*	−0.04	0.06	0.14 *	0.06	<0.01	0.06	−0.03	0.06	0.01	0.06	<0.01	0.06
*Fear of maturity*	0.12 **	0.03	0.03	0.04	−0.06	0.04	−0.03	0.04	−0.03	0.04	−0.05	0.04
*Asceticism*	0.09	0.05	<0.01	0.05	< 0.01	0.05	0.05	0.05	<0.01	0.05	0.05	0.05
*Impulsiveness*	0.17 **	0.05	0.03	0.05	0.13 *	0.05	0.11	0.05	0.18 **	0.05	0.06	0.05
*Social insecurity*	<0.01	0.05	0.07	0.05	0.02	0.06	0.08	0.06	0.05	0.05	0.12 *	0.05
Fear of loneliness			0.08	0.04	0.03	0.04	0.03	0.04	0.04	0.04	0.02	0.04
Social withdrawal			0.06	0.04	0.07	0.04	0.03	0.04	0.07	0.04	0.04	0.04
Fear of loneliness X Social withdrawal			0.15 ***	0.03	0.03	0.03	0.07 *	0.03	0.06	0.03	0.12 **	0.03
*r* ^2^	0.21 ***		0.07 ***		0.05 **		0.04 **		0.06 ***		0.05 **	

Note. * *p* < 0.05, ** *p* < 0.01, *** *p* < 0.001.

**Table 4:** Moderated indirect effects of ineffectiveness, fear of maturity and impulsiveness on physical, psychological humiliation–coercion and social received violence through fear of loneliness.

		Dependent Variables (Received Violence)					
		Physical		Psychological Humiliation–Coercion		Social	
Independent variables	Level of the moderator	*z*	*p*	*z*	*p*	*z*	*p*
Ineffectiveness	Low social withdrawal	−1.65	0.099	−1.03	0.302	−1.76	0.078
	Average social withdrawal	1.70	0.089	0.74	0.454	0.66	0.507
	High social withdrawal	2.81 *	0.005	1.86	0.062	2.34 *	0.019
Fear of maturity	Low social withdrawal	−1.60	0.109	−1.02	0.308	−1.71	0.088
	Average social withdrawal	1.65	0.099	0.74	0.456	0.66	0.509
	High social withdrawal	2.61 **	0.009	1.80	0.071	2.22 *	0.026
Impulsiveness	Low social withdrawal	−1.61	0.106	−1.02	0.306	−1.72	0.085
	Average social withdrawal	1.66	0.096	0.74	0.456	0.66	0.508
	High social withdrawal	2.66 **	0.008	1.82	0.068	2.25 *	0.024

Note. * *p* < 0.05, ** *p* < 0.01.

## Data Availability

The datasets generated during and/or analysed during the current study are available from the corresponding author on reasonable request.
